# A hematopoietic contribution to microhemorrhage formation during antiviral CD8 T cell-initiated blood-brain barrier disruption

**DOI:** 10.1186/1742-2094-9-60

**Published:** 2012-03-27

**Authors:** Holly L Johnson, Yi Chen, Georgette L Suidan, Jeremiah R McDole, Anne K Lohrey, Lisa M Hanson, Fang Jin, Istvan Pirko, Aaron J Johnson

**Affiliations:** 1 Departments of Neurology, Mayo Clinic, Rochester, MN, USA; 2 Departments of Immunology, Mayo Clinic, Rochester, MN, USA; 3 Departments of Neurology, University of Cincinnati College of Medicine, Cincinnati, OH, USA; 4 Departments of Neuroscience, University of Cincinnati College of Medicine, Cincinnati, OH, USA; 5 Departments of Immunology and Neurology, Mayo Clinic, Rochester, MN 55902, USA

**Keywords:** Blood-brain barrier, CD8 T cell, Hematopoietic factors, Hemorrhage, MRI, Theiler's murine encephalomyelitis

## Abstract

**Background:**

The extent to which susceptibility to brain hemorrhage is derived from blood-derived factors or stromal tissue remains largely unknown. We have developed an inducible model of CD8 T cell-initiated blood-brain barrier (BBB) disruption using a variation of the Theiler's murine encephalomyelitis virus (TMEV) model of multiple sclerosis. This peptide-induced fatal syndrome (PIFS) model results in severe central nervous system (CNS) vascular permeability and death in the C57BL/6 mouse strain, but not in the 129 SvIm mouse strain, despite the two strains' having indistinguishable CD8 T-cell responses. Therefore, we hypothesize that hematopoietic factors contribute to susceptibility to brain hemorrhage, CNS vascular permeability and death following induction of PIFS.

**Methods:**

PIFS was induced by intravenous injection of VP2_121-130 _peptide at 7 days post-TMEV infection. We then investigated brain inflammation, astrocyte activation, vascular permeability, functional deficit and microhemorrhage formation using T2*-weighted magnetic resonance imaging (MRI) in C57BL/6 and 129 SvIm mice. To investigate the contribution of hematopoietic cells in this model, hemorrhage-resistant 129 SvIm mice were reconstituted with C57BL/6 or autologous 129 SvIm bone marrow. Gadolinium-enhanced, T1-weighted MRI was used to visualize the extent of CNS vascular permeability after bone marrow transfer.

**Results:**

C57BL/6 and 129 SvIm mice had similar inflammation in the CNS during acute infection. After administration of VP2_121-130 _peptide, however, C57BL/6 mice had increased astrocyte activation, CNS vascular permeability, microhemorrhage formation and functional deficits compared to 129 SvIm mice. The 129 SvIm mice reconstituted with C57BL/6 but not autologous bone marrow had increased microhemorrhage formation as measured by T2*-weighted MRI, exhibited a profound increase in CNS vascular permeability as measured by three-dimensional volumetric analysis of gadolinium-enhanced, T1-weighted MRI, and became moribund in this model system.

**Conclusion:**

C57BL/6 mice are highly susceptible to microhemorrhage formation, severe CNS vascular permeability and morbidity compared to the 129 SvIm mouse. This susceptibility is transferable with the bone marrow compartment, demonstrating that hematopoietic factors are responsible for the onset of brain microhemorrhage and vascular permeability in immune-mediated fatal BBB disruption.

## Introduction

Unregulated permeability of the blood-brain barrier (BBB) is a feature of many neurological diseases as diverse as stroke, viral hemorrhagic fevers, HIV dementia, shock, cerebral malaria, multiple sclerosis, acute hemorrhagic leukoencephalitis and epilepsy [1-16]. An impermeable BBB is a major obstacle to effective therapeutic delivery of chemotherapy [17]. There is increasing evidence that inflammatory mediators expressed by central nervous system (CNS) infiltrating immune cells contribute to BBB permeability [6,18-20]. The mechanism by which blood-derived factors and tissue stromal cells contribute to CNS vascular permeability *in vivo *remain undefined, however, indicating that inflammation-driven BBB permeability is a complex condition. For all the above reasons, developing model systems to define inflammatory mediators involved in susceptibility and resistance to BBB disruption could ultimately lead to the discovery of novel therapeutics designed to both intervene with and augment CNS vascular permeability.

Clinical observations and rodent models support a role for inflammatory immune cells in promoting opening of the BBB. Monocytes and neutrophils have been demonstrated to contribute to CNS vascular permeability [21]. CD4 T-cell subsets have also been implicated in BBB disruption, including the demonstration of tight junction protein alterations that are initiated by TH_17 _cells [22,23]. Our studies have defined antigen-specific CD8 T cells as an additional potent mediator of rapid BBB disruption [24-27]. The concept that CD8 T cells and high antigenic peptide load promote an important link between inflammatory disease and vascular permeability is further supported by numerous studies in cerebral malaria and viral hemorrhagic fevers [13,16,28-33]. In addition, CD8 T cells are a major lymphocyte subset observed in multiple sclerosis lesions characterized by the presence of gadolinium enhancement visible by magnetic resonance imaging (MRI) [34,35]. However, the extent to which these different immune cell types utilize common mechanisms to promote BBB disruption remains unknown. The extent to which susceptibility to brain hemorrhage is due to blood-derived factors or stromal tissue also remains unknown.

Our model of CD8 T-cell-initiated BBB disruption utilizes a variation of the Theiler's murine encephalomyelitis virus (TMEV) model commonly used to study multiple sclerosis [24-27]. During acute TMEV infection, 50% to 70% of CNS-infiltrating CD8 T cells in the C57BL/6 mouse are specific for an immunodominant virus peptide, VP2_121-130 _(FHAGSLLVFM), presented in the context of the D^b ^class I molecule. The peak expansion of D^b^: VP2_121-130 _epitope-specific CD8 T cells is 7 days postinfection and is predominantly limited to the CNS [36]. Intravenous injection of VP2_121-130 _peptide at 7 days post-TMEV infection results in activation of astrocytes and microglia, alteration of tight junction proteins, extensive CNS vascular permeability and functional deficit in the C57BL/6 mouse [24-27]. Vascular permeability is perforin-dependent and exclusive to the CNS, the site of D^b^: VP2_121-130 _epitope-specific CD8 T cell expansion [24,25]. In this model, BBB disruption is followed by death approximately 48 to 72 hours later. We have termed this condition "peptide-induced fatal syndrome" (PIFS).

The PIFS model is inducible with administration of VP2_121-130 _peptide, enabling the study of early and late gene expression events during immune-mediated BBB disruption in the C57BL/6 mouse strain. In contrast to C57BL/6 mice, 129 SvIm mice are resistant to PIFS, despite this strain's having an indistinguishable CNS-infiltrating D^b^: VP2_121-130 _epitope-specific CD8 T-cell response. In this study, we evaluated the differential effects of CD8 T cells in initiating CNS vascular permeability in these two strains. We also employed the PIFS model system to address the extent to which blood-derived factors or CNS stromal tissues contribute to functional deficit, microhemorrhage formation and CNS vascular permeability.

## Methods

### Animals

The mouse strains C57BL/6 (strain 000664; The Jackson Laboratory, Bar Harbor, ME, USA) and 129 SvIm (strain 002448; The Jackson Laboratory) were used in this study because of their differential susceptibility to [27]. All mice were obtained at 6 weeks of age. All experiments were approved by the Institutional Animal Care and Use Committees of the University of Cincinnati and the Mayo Clinic.

### *In vitro *cytotoxic T lymphocyte assay

C57SV cells transfected with the genes encoding the TMEV protein VP2 were used as targets, as previously described [37]. A standard 4-hour ^51^Cr release assay was done to determine cytotoxic T lymphocyte (CTL) activity in CNS-infiltrating lymphocyte preparations obtained from C57BL/6 and 129 SvIm mice. Untransfected and VP2 gene-transfected C57SV cells were labeled with ^51^Cr for 2 hours. Freshly isolated effector cells were incubated with labeled target cells for 4 hours. The percentage specific lysis of target cells was calculated from released ^51^Cr as follows: 100 × (experimental release - spontaneous release)/(total release in detergent - spontaneous release). The number of effector T cells added was estimated by counting the cells with lymphocyte morphology by microscopic inspection.

### Virus infection and induction of central nervous system vascular permeability

CNS vascular permeability was induced as described previously [27]. Briefly, all mice were intracranially infected with 2 × 10^6 ^plaque-forming units of Daniel's strain of TMEV. Seven days post-TMEV infection mice were injected intravenously with 0.1 mg of VP2_121-130 _(FHAGSLLVFM) to induce CNS vascular permeability or mock control E7 (RAHYNIVTF) peptide (GenScript, Piscataway, NJ, USA).

### Flow cytometry

Seven days post-TMEV infection, brain-infiltrating lymphocytes were isolated from mouse brains by collagenase digestion and a Percoll gradient as previously described [38]. Allophycocyanin (APC) D^b^: VP2_121-130 _tetramer and APC D^b^: E7 tetramer were constructed as previously described and used in conjunction with anti-CD8-fluorescein isothiocyanate (FITC) (553031; BD Pharmingen, San Diego, CA, USA) and anti-CD45 (557235; BD Pharmingen) [23]. Neutrophils were assessed by staining with anti-Ly6G antibody (127603; BioLegend, San Diego, CA, USA) conjugated with phycoerythrin-streptavidin (Invitrogen, Carlsbad, CA, USA). Samples were read on a BD LSR II Flow Cytometer (BD Biosciences, San Jose, CA, USA) and analyzed with BD FACSDiva version 6.0 software (BD Biosciences).

### Rotarod

Animals were placed on the Rotamex-5 rotarod apparatus (Columbus Instruments, Columbus, OH, USA), with the speed increased from 5 to 40 rpm over 7 minutes as previously described [39]. Animals were trained twice daily for 3 days. On the final day, a baseline ability in seconds remaining on the rotarod was determined for each mouse. Twenty-four hours following administration of VP2_121-130 _peptide, a rotarod score in seconds was determined. Functional ability was represented as percentage initial ability on the rotarod using the formula: 100% × (seconds on rotarod prior to VP2 peptide administration)/(seconds on rotarod 24 hours post-VP2 peptide administration).

### Western blot analysis

Brain tissue samples were lysed in radioimmunoprecipitation assay buffer [25] (10 mmol/L Tris, 140 mmol/L NaCl, 1% Triton X-100, 1% Na^+ ^deoxycholate, 0.1% SDS and protease inhibitor cocktail (78410; Pierce Biotechnology, Rockford, IL, USA), pH 7.5) and centrifuged for 15 minutes at 10,000 rpm. Samples were normalized for protein content after protein concentration was assessed using a bicinchoninic acid protein assay (23223; Pierce Biotechnology). For glial fibrillary acidic protein (GFAP) detection, 10 μg of protein per well were loaded onto 4% to 20% precise protein gels (25244; Pierce Biotechnology) with BupH Tris-4-(2-hydroxyethyl)-1-piperazineethanesulfonic acid-SDS running buffer (28398; Pierce Biotechnology). Gels were transferred onto Immun-Blot LF polyvinylidene fluoride membranes (162-0177; Bio-Rad Laboratories, Hercules, CA, USA) using Tris transfer buffer (400 mmol/ml Tris base, 70 mmol/ml glycine and 10% methanol). Mouse anti-GFAP (1:1,000 dilution, 556329; BD Pharmingen) and goat anti-mouse immunoglobulin G conjugated to horseradish peroxidase were used to detect GFAP (A3682; Sigma-Aldrich, St Louis, MO, USA). Western blot films were analyzed by densitometry using Scion Image software (Scion Corp, Frederick, MD, USA). Background was subtracted from each band, and data are expressed in arbitrary units.

### Fluorescein isothiocyanate-albumin permeability assay

Mice were injected intravenously with 10 mg of FITC-albumin (A9771; Sigma-Aldrich) 23 hours after VP2_121-130 _peptide administration to induce PIFS. Brains were harvested 1 hour after administration of FITC-albumin and frozen on aluminum foil on dry ice. The right hemisphere was homogenized with radioimmunoprecipitation assay buffer as described above using Western blot methodology. Homogenates were read on a fluorescent plate reader at 488-nm excitation and 525-nm emission to detect FITC-albumin leakage into the brain. Data were collected using SpectraMax software (Molecular Devices, Sunnyvale, CA, USA).

### Bone marrow transplants

To determine the contribution of C57BL/6 hematopoietic cells to susceptibility to PIFS and microhemorrhage formation, 8-week-old male 129 SvIm mice were lethally irradiated with two pulses of 700 and 425 rad 4 hours apart. Twenty-four hours later 129 Svlm mice were intravenously reconstituted with 10^7 ^autologous bone marrow or C57BL/6 Ly5.1^+ ^bone marrow-derived cells. Eight weeks postreconstitution, mice were infected with TMEV. Seven days post-TMEV infection, animals were administered VP2_121-130 _peptide and analyzed for susceptibility to PIFS. A similar protocol was followed to determine the contribution of C57BL/6 hematopoietic cells to susceptibility to functional deficit and CNS vascular permeability, except that 129 Svlm mice were reconstituted with 10^7 ^autologous bone marrow or C57BL/6 Ly5.1^+ ^bone marrow-derived cells at 5 weeks of age and infected with TMEV at 4 weeks postreconstitution. Animals were administered VP2_121-130 _peptide 7 days post-TMEV infection.

### Magnetic resonance imaging

*In vivo *MRI was performed in a 7-Tesla narrow-bore small animal imaging system (Bruker BioSpin, Billerica, MA, USA). Inhalational isoflurane anesthesia was used. A custom-made saddle coil was employed for acquisition and excitation. We acquired three-dimensional, T2*-weighted images (gradient echo fast imaging pulse sequence: repetition time (TR) = 150 ms, echo time (TE) = 10 ms, flip angle = 15°, field of view = 4 × 2.5 × 2.5 cm, matrix: 256 × 128 × 128, number of excitations = 4). MRI scans were analyzed using Analyze (Biomedical Imaging Resource, Mayo Clinic, Rochester, MN, USA) and ImageJ software (National Institutes of Health, Bethesda, MD, USA). A blinded technician using Analyze captured axial slices from each animal. Axial slices representing *z *= 0 according to stereotactic coordinate criteria put forward by Kovacevic *et al. *[40] were obtained using Analyze. These axial slices were then analyzed using ImageJ software to enable threshold analysis and densitometric analysis. Only T2*-weighted MRI lesions obtained from animals not representative of common brain structures (that is, ventricles) were quantified. Once data were obtained, the total T2*-weighted MRI lesion load as a percentage of whole brain was determined for each axial slice. Samples were then unblinded, and the T2*-weighted MRI lesion loads among animals in the different experimental groups were determined. Additionally, a Bruker Avance II 7-Tesla vertical bore small-animal MRI system (Bruker BioSpin) was used to acquire T1-weighted images to evaluate CNS vascular permeability. Image acquisition was performed as described previously [26,41]. Mice were given an intraperitoneal injection of gadolinium using weight-based dosing of 100 mg/kg. After a standard delay of 15 minutes, a volume acquisition T1-weighted spin-echo sequence was used (TR = 300 ms, TE = 9.5 ms, FOV = 32 × 19.2 × 19.2 mm, matrix = 192 × 96 × 96, number of averages = 1) to obtain T1-weighted images. Analyze 10 software (Biomedical Imaging Resource, Mayo Clinic) was used to quantify the three-dimensional volume of vascular permeability as previously described [42-44]. The three-dimensional volume extractor tool was employed to extract brains from the gadolinium-enhanced, T1-weighted images. Areas of gadolinium leakage were defined using semiautomated methods in the three-dimensional region of interest tool. The identified volume of gadolinium enhancement was saved as a three-dimensional object, then the three-dimensional sampling tool was used to calculate the actual volume of this object. Figures were generated by performing three-dimensional object rendering using the volume-rendering tool to visualize the volume of contrast enhancement.

### Statistical analysis

Mean and standard error values for rotarod scores, FITC-albumin quantification, Western blot quantification and quantification of the three-dimensional volume of gadolinium leakage from vasculature were calculated using the SigmaStat software program (SYSTAT Software Inc, Chicago, IL, USA). Bar graphs with standard error values were plotted using the SigmaPlot software program (SYSTAT Software Inc). To determine the significance level between groups, a Student's *t*-test was performed between E7 and VP2_121-130 _within the same strain using SigmaPlot software. A Student's *t*-test was also performed to determine the significance level between the bone marrow transplant groups.

## Results

### Comparable D^b^: VP2_121-130 _epitope dominance among CD8 T cells and neutrophil recruitment in C57BL/6 and 129 SvIm mouse strains

**Figure 1:**
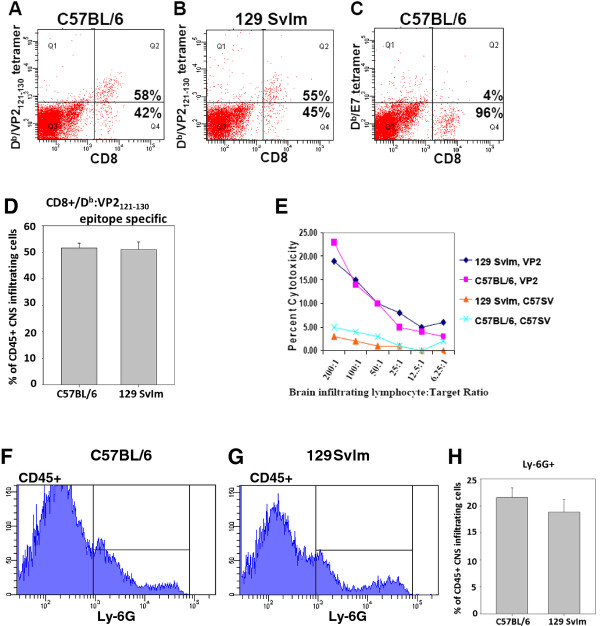
**D^b^: VP2_121-130 _epitope dominance among CNS-infiltrating CD8 T cells and neutrophil infiltration during acute TMEV infection**. Central nervous system (CNS)-infiltrating lymphocytes were isolated 7 days post-Theiler's murine encephalomyelitis virus (TMEV) infection. Shown is fluorescence-activated cell-sorting analysis staining for CD8 and D^b^: VP2_121-130 _peptide tetramer among CNS-infiltrating lymphocytes isolated from a representative **(A) **C57BL/6 mouse or **(B) **129 SvIm mouse. **(C) **CNS-infiltrating C57BL/6 lymphocytes stained for CD8 and negative control D^b^: E7 peptide tetramer. **(D) **C57BL/6 and 129 Svlm mice display comparable levels of brain-infiltrating CD45^+ ^cells that are CD8^+ ^and specific for the D^b^: VP2_121-130 _epitope. **(E) **Equivalent capacity of CNS-infiltrating cytotoxic T lymphocytes isolated from TMEV-infected C57BL/6 and 129 SvIm mice to utilize perforin to kill VP2-transfected C57SV target cells in a chromium release assay. Untransfected C57SV cells served as a negative control. CD45^hi ^population was gated and analyzed for the percentage of Ly6G^+ ^cells in both **(F) **C57BL/6 and **(G) **129 Svlm mice (*n *= 5 per group). **(H) **Bar chart showing that C57BL/6 and 129 Svlm mice display comparable levels of Ly6G^+ ^cells, which are indicative of being a neutrophil subset. In 1 million isolated total cells recorded by flow cytometry, there were no statistically significant differences in CD45^+^, Ly6G^+^, or CD8^+ ^D^b^: VP2_121-130 _tetramer-positive cells collected (data not shown).

Seven days post-intracranial infection with TMEV, C57BL/6 mice developed a robust CNS-infiltrating CD8 T-cell response toward the virus peptide VP2_121-130 _presented in the context of the D^b ^class I molecule. Using D^b^: VP2_121-130 _peptide major histocompatibility complex (MHC) tetramers, we determined that 50% to 70% of CNS-infiltrating CD8 T cells are specific for the D^b^: VP2_121-130 _epitope (Figures 1A and 1D) [36,39]. Similar to C57BL/6 mice, 129 SvIm mice displayed comparable levels of epitope dominance, including a similar percentage of CD45^+ ^inflammatory infiltrate (Figures 1B and 1D). Figure 1C depicts a representative D^b^: E7 peptide MHC tetramer-negative staining control. CNS-infiltrating lymphocytes isolated from both strains exhibited comparable levels of perforin-mediated cytotoxic killing at 7 days post-TMEV infection (Figure 1E). Neutrophil infiltration as measured by anti-Ly6G staining was also similar between these two strains (*n *= 5 per strain) (Figures 1F through 1H). In 1,000,000 events recorded, we did not observe statistically significant differences in CNS-infiltrating CD45^+^, Ly6G^+^, CD8^+^, or CD8^+ ^D^b^: VP2_121-130 _tetramer-positive cells isolated from the brains of C57BL/6 and 129 SvIm mice (Figure 1; data not shown). These data demonstrate that both of these mouse strains mount a similar and functional D^b^: VP2_121-130 _epitope-specific CD8 T-cell-mediated response to TMEV infection.

### Functional deficit in C57BL/6 but not 129 SvIm mice following induction of peptide-induced fatal syndrome

**Figure 2:**
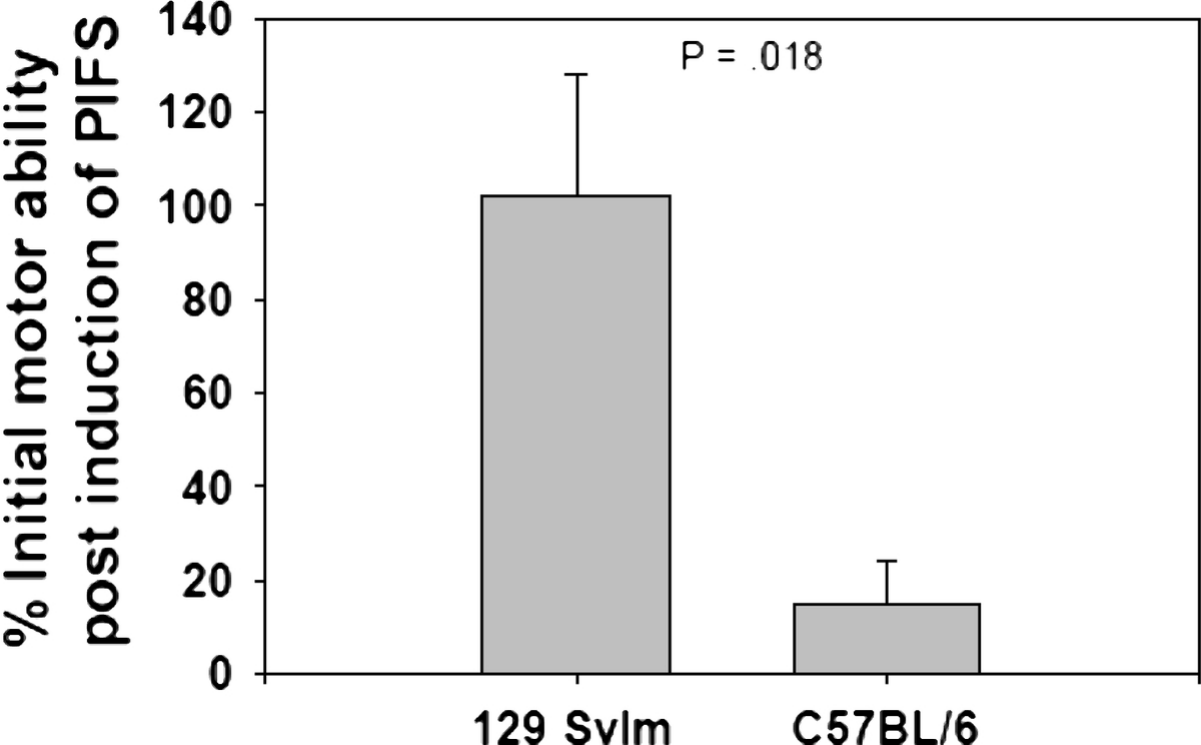
**C57BL/6 mice with peptide-induced fatal syndrome are unable to negotiate the rotarod**. Seven-day Theiler's murine encephalomyelitis virus-infected C57BL/6 and 129 SvIm mice were intravenously administered VP2_121-130 _peptide. Rotarod performance is plotted as the percentage of initial rotarod score prior to VP2_121-130 _peptide administration (*n *= 16 for each group).

We previously published data showing that 7-day TMEV-infected C57BL/6 mice that are intravenously administered the VP2_121-130 _peptide epitope during the peak of the VP2_121-130 _CD8 T-cell response succumb to a fatal syndrome characterized by extensive CNS vascular permeability and microhemorrhage formation [24-27]. In contrast, the 129 SvIm mouse strain, despite having comparable expansion of D^b^: VP2_121-130 _epitope-specific CD8 T cells in the CNS (Figure 1), is resistant to this fatal syndrome. To quantify the severe functional deficits that develop in mice with PIFS, we assessed rotarod performance. At 24 hours after induction of PIFS, C57BL/6 mice (*n *= 16) ran at 12% of their initial ability. By comparison, 129 SvIm mice 24 hours after administration of VP2_121-130 _peptide (*n *= 16) negotiate the rotarod at their initial ability level (Figure 2). This experiment demonstrates that, despite mounting a comparable CD8 T-cell response restricted toward the D^b^: VP2_121-130 _epitope (Figure 1), only C57BL/6 mice develop severe functional deficit that ultimately proceeds to the animals' becoming moribund.

### Enhanced astrocyte glial fibrillary acidic protein expression in C57BL/6 mice, but not in 129 SvIm mice, following administration of VP2_121-130 _peptide

**Figure 3:**
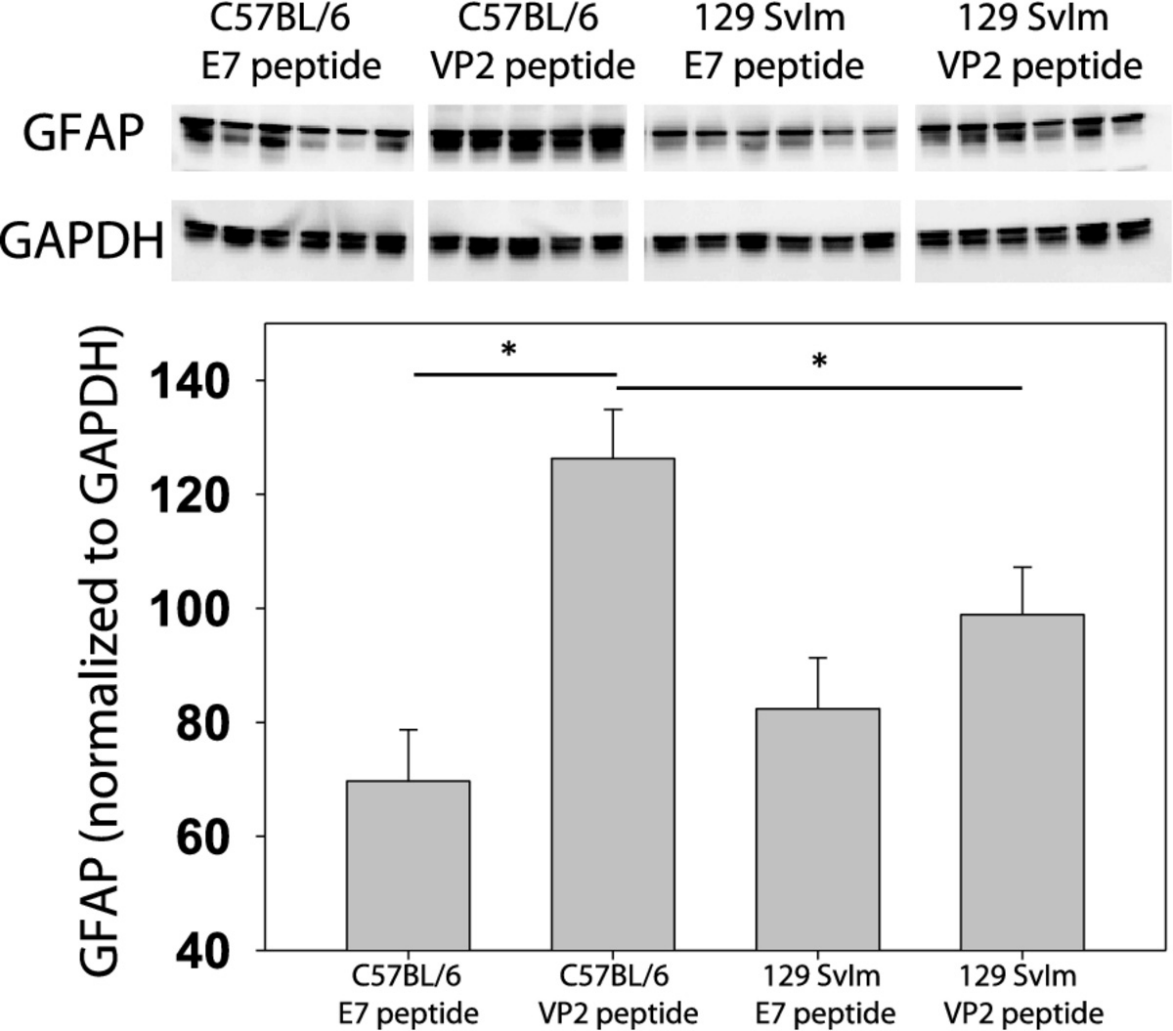
**Activation of an astrocyte stress response in the brain accompanies peptide-induced fatal syndrome as determined by quantifiable glial fibrillary acidic protein Western blot analysis**. C57BL/6 and 129 SvIm strain animals were infected for 7 days intracranially with Theiler's murine encephalomyelitis virus prior to systemic treatment with VP2_121-130 _peptide to induce peptide-induced fatal syndrome. Twenty-four hours after peptide treatment the right hemispheres of mice were homogenized and analyzed for glial fibrillary acidic protein (GFAP) levels. Shown are protein levels and normalization of GFAP to glyceraldehyde 3-phosphate dehydrogenase as a loading control. Asterisks denote statistical significance (*P *< 0.05) between treatment groups. Each group consisted of six mice. One VP2_121-130 _peptide-treated C57BL/6 mouse had to be killed and was removed from the study.

Astrocyte activation, as measured by GFAP expression, colocalizes with vascular permeability in C57BL/6 mice with PIFS [24]. To determine the extent to which astrocytes were activated in C57BL/6 and 129 SvIm mice in the PIFS model, we evaluated (by quantitative Western blot analysis) GFAP levels in animals that had been administered E7 or VP2_121-130 _peptide (*n *= 6 per group). In Figure 3, we present significantly upregulated GFAP expression in VP2_121-130 _peptide-treated C57BL/6 mice with PIFS compared to mock E7 peptide-treated controls. GFAP expression in VP2_121-130 _peptide-treated C57BL/6 mice was also significantly higher than that in 129 SvIm mice that were administered either E7 or VP2_121-130 _peptide (Figure 3). This experiment demonstrated that, in addition to becoming moribund, C57BL/6 mice induced to develop PIFS had significantly higher GFAP expression, which is indicative of higher levels of astrocyte activation, than 129 SvIm mice did.

### C57BL/6 mice have increased central nervous system vascular permeability compared to 129 SvIm mice upon induction of peptide-induced fatal syndrome

**Figure 4:**
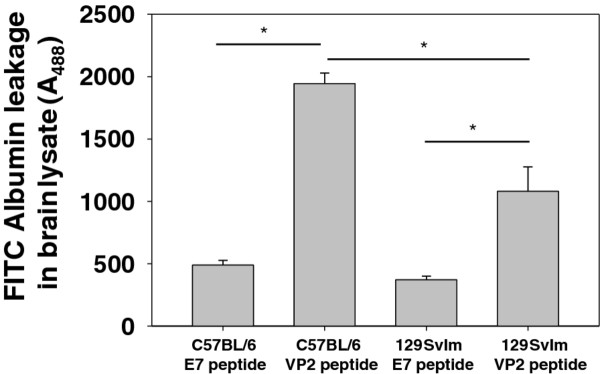
**Increased vascular permeability in the brains of C57BL/6 compared to 129 SvIm mice following induction of peptide-induced fatal syndrome**. Intravenously administered fluorescein isothiocyanate (FITC)-albumin is detectable in homogenized brains of C57BL/6 mice 24 hours after administration of VP2_121-130_, but not E7 peptide. VP2_121-130 _peptide-treated 129 SvIm mice have detectable FITC-albumin leakage compared to E7 peptide-treated controls. C57BL/6 mice have significantly higher levels of detectable FITC-albumin in the forebrain than 129 SvIm mice 24 hours after induction of peptide-induced fatal syndrome. Asterisks denote statistical significance (*P *< 0.05). Each group consisted of six mice. One VP2_121-130 _peptide-treated C57BL/6 mouse had to be killed and was removed from the study.

Seven-day TMEV-infected C57BL/6 mice administered VP2_121-130 _peptide to induce PIFS have high levels of CNS vascular permeability [24]. To determine the extent to which C57BL/6 mice have increased CNS vascular permeability compared to 129 SvIm mice, we infected these two strains with TMEV. Seven days later we administered mock E7 peptide or VP2_121-130 _peptide to induce PIFS (*n *= 6 per group). Animals were injected with FITC-albumin 23 hours after administration of mock E7 peptide or VP2_121-130 _peptide and allowed to circulate for 1 hour. Brains were then harvested, homogenized and measured for permeability of FITC-albumin into the CNS according to our previously described methods [24,25]. As shown in Figure 4, VP2_121-130 _peptide-treated C57BL/6 and 129 SvIm mice had increased FITC-albumin leakage compared to mock E7 peptide-treated controls. However, significantly higher permeability was observed in C57BL/6 mice compared to 129 SvIm mice after both had received VP2_121-130 _peptide administration. Additionally, we have previously described that vascular endothelial growth factor (VEGF) contributes to vascular permeability in C57BL/6 mice following administration of VP2_121-130 _peptide [25]. We therefore assessed VEGF levels using Western blot analysis and found that there was a significant increase in VEGF among 7-day TMEV-infected C57BL/6 mice administered VP2 _121-130 _peptide compared to C57BL/6 mice administered mock E7 peptide (*P *= 0.045). However, there was no significant difference between 7-day TMEV-infected 129 Svlm mice administered VP2_121-130 _or E7 peptide (*P *= 0.998) (data not shown). This increase in VEGF in VP2_121-130_-treated C57BL/6 mice may contribute to the significantly higher vascular permeability seen in these mice, as previously described [25].

### C57BL/6 mice with peptide-induced fatal syndrome have greater microhemorrhage formation than 129 SvIm mice as measured by T2*-weighted magnetic resonance imaging

**Figure 5:**
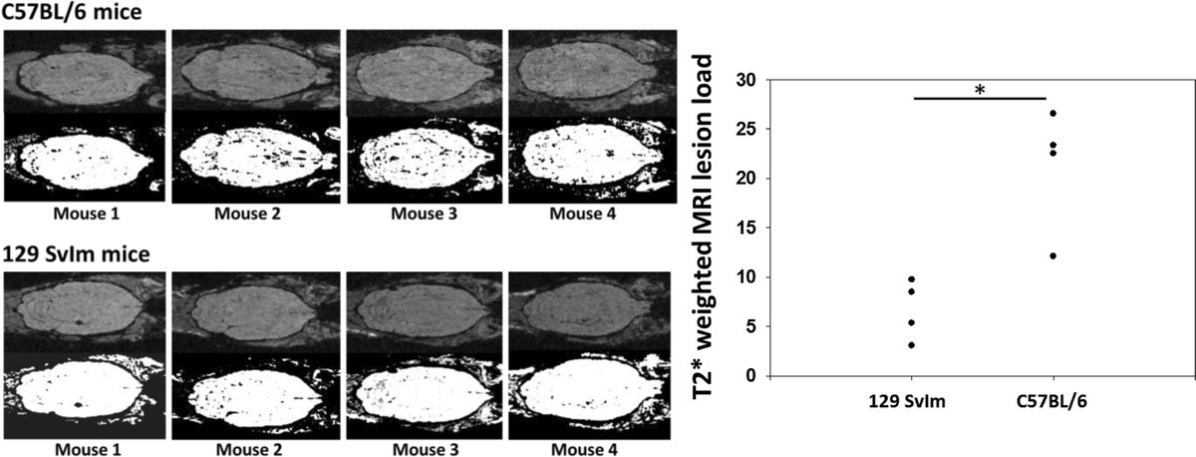
Microhemorrhage formation in C57BL/6 mice is observable by T2*-weighted MRI (top panels). Permeability-resistant 129 SvIm mice (bottom panels) present with reduced microhemorrhages compared to C57BL/6 mice. Raw magnetic resonance imaging scans are shown above images analyzed using ImageJ software. Pixel value analysis revealed significantly higher microhemorrhages in C57BL/6 mice than in the 129 SvIm strain (*n *= 4 in each group, *P *= 0.003). ImageJ software was used to analyze threshold of T2*-weighted MRI lesions.

C57BL/6 mice present with higher CNS vascular permeability than 129 SvIm mice, which may be dependent on increased VEGF expression. Nevertheless, the observation that both C57BL/6 and 129 SvIm mice have measurable increases in CNS vascular permeability as measured by FITC-albumin leakage following administration of VP2_121-130 _peptide was intriguing. To better understand the nature of CNS vascular permeability in these two strains, we evaluated the PIFS model by utilizing MRI. We previously demonstrated histologically that C57BL/6 mice with PIFS present with extensive microhemorrhage formation throughout the brain [27]. We also reported previously that these microhemorrhages can be scanned with T2*-weighted MRI and that CNS vascular permeability occurs in areas of hemorrhage [26]. We therefore determined the extent to which brain mircrohemorrhage had occurred in the CNSs of these two strains of mice when they were administered VP2_121-130 _peptide 7 days post-TMEV infection to induce PIFS (*n *= 4 per group). As shown in Figure 5, 24 hours after administration of VP2_121-130 _peptide, C57BL/6 mice presented with a large number of punctuate microhemorrhages throughout the brain, consistent with our previously published observations. We also previously found that E7 mock peptide-treated control animals do not present with these microhemorrhages, demonstrating that the formation of lesions is dependent on administration of VP2_121-130 _peptide [24-27]. In contrast, 129 SvIm mice administered VP2_121-130 _peptide had markedly less detectable microhemorrhages as measured by T2*-weighted MRI (*P *= 0.003) (Figure 5). Therefore, consistent with the 129 SvIm strain's having lower astrocyte activation, FITC-albumin leakage and morbidity, this strain also presents with reduced microhemorrhage formation following induction of PIFS with administration of VP2_121-130 _peptide.

### A hematopoietic factor contributes to susceptibility of C57BL/6 mice to peptide-induced fatal syndrome

**Figure 6:**
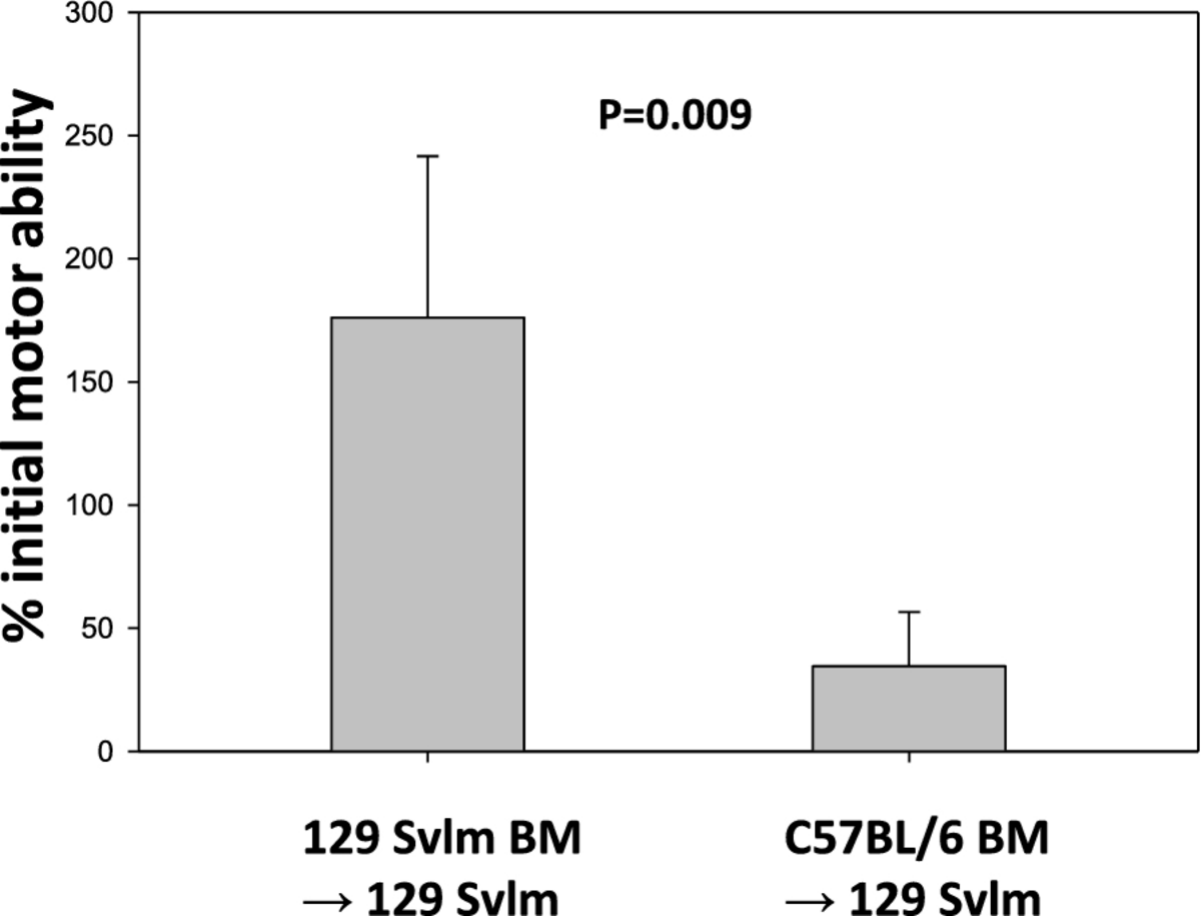
**129 Svlm mice reconstituted with C57BL/6 bone marrow are unable to negotiate the rotarod**. Irradiated 129 Svlm mice were reconstituted with either autologous 129 Svlm bone marrow (*n *= 8) or C57BL/6 bone marrow (*n *= 10). Both groups were intravenously administered VP2_121-130 _peptide 7 days post-Theiler's murine encephalomyelitis virus infection. Rotarod performance is plotted as the percentage of initial rotarod score prior to VP2_121-130 _peptide administration.

We determined that C57BL/6 mice are susceptible to PIFS, whereas 129 SvIm mice are resistant to it. This demonstrates that genetic background determines susceptibility to this condition. To determine the extent to which hematopoietic factors contributed to strain-related susceptibility to PIFS, lethally irradiated, resistant 129 Svlm mice were reconstituted with bone marrow from susceptible C57BL/6 mice (*n *= 10). 129 Svlm mice reconstituted with autologous 129 Svlm bone marrow (*n *= 8) were used as negative controls. Four weeks postreconstitution, animals were intracranially infected with TMEV. On day 7 postinfection, animals were intravenously administered VP2_121-130 _peptide and assessed for the onset of PIFS as measured by functional deficit through rotarod analysis. 129 Svlm mice reconstituted with C57BL/6 bone marrow became susceptible to moribundity following induction of PIFS as measured by their inability to negotiate the rotarod. 129 Svlm mice that received autologous 129 Svlm bone marrow remained resistant to PIFS (Figure 6). This experiment demonstrated that a hematopoietic factor associated with the C57BL/6 mouse genetic background contributed to susceptibility to PIFS.

### A hematopoietic factor contributes to enhanced CD8 T-cell-initiated astrocyte glial fibrillary acidic protein expression, microhemorrhage formation, and CNS vascular permeability following induction of peptide-induced fatal syndrome

**Figure 7:**
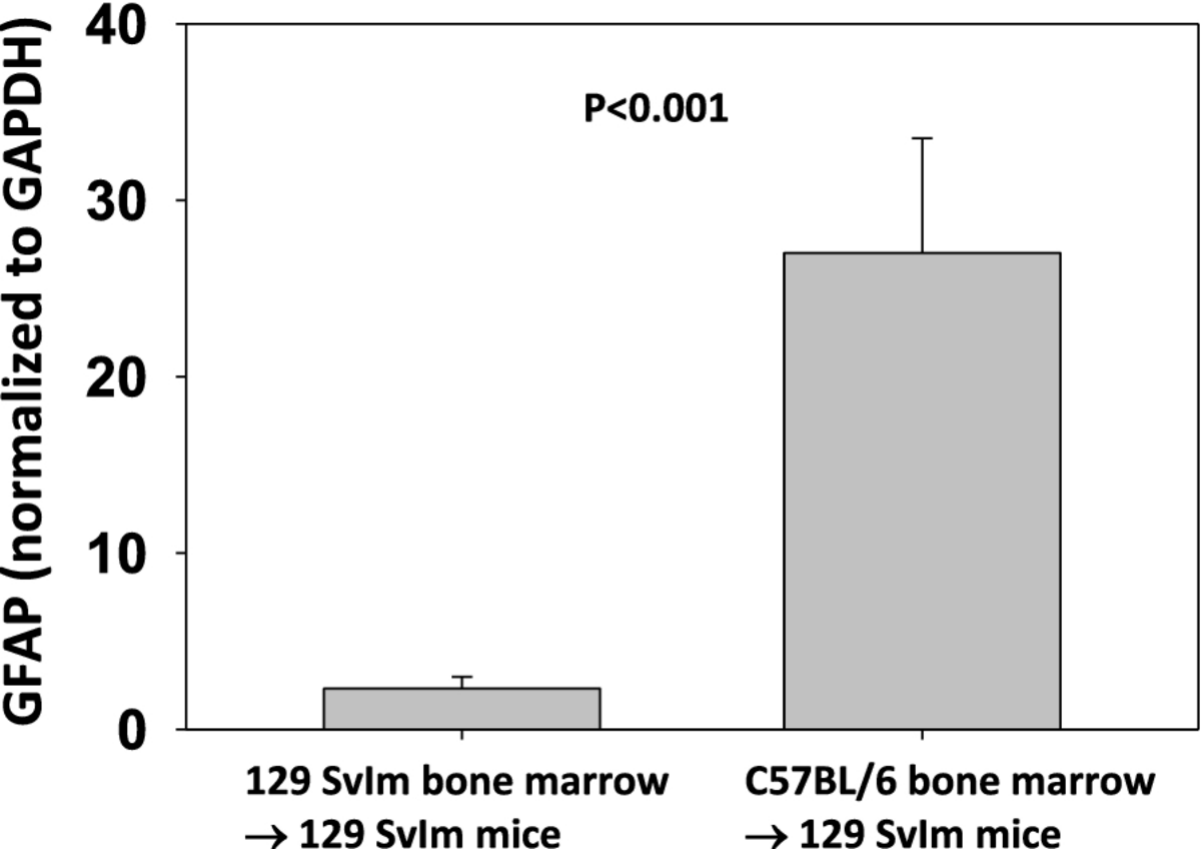
**A hematopoietic factor contributes to enhanced astrocyte glial fibrillary acidic protein expression**. Irradiated 129 Svlm mice were reconstituted with either autologous 129 Svlm bone marrow (*n *= 12) or C57BL/6 bone marrow (*n *= 5). Both groups were intravenously administered VP2_121-130 _peptide 7 days post-Theiler's murine encephalomyelitis virus infection. 129 Svlm mice reconstituted with C57BL/6 bone marrow displayed a significant increase in glial fibrillary acidic protein expression in brain lysates compared to 129 Svlm mice that received syngeneic bone marrow transfer (*P *< 0.001).

**Figure 8:**
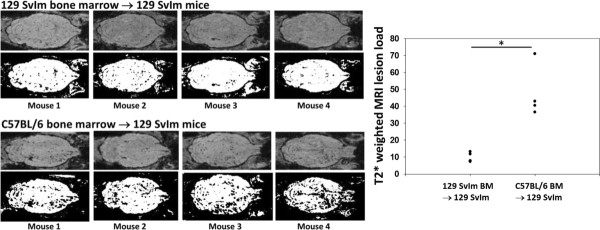
**A hematopoietic factor contributes to enhanced CD8 T-cell-induced microhemorrhage formation**. The top panels show individual 129 SvIm mice receiving autologous bone marrow. Meanwhile, 129 SvIm mice reconstituted with C57BL/6 mouse bone marrow display significantly increased microhemorrhage formation as measured by T2*-weighted magnetic resonance imaging 24 hours post-VP2_121-130 _peptide administration to induce peptide-induced fatal syndrome (bottom panels). ImageJ software was used to analyze the threshold value of T2*-weighted MRI lesions (*n *= 4 in each group, *P *= 0.003).

**Figure 9:**
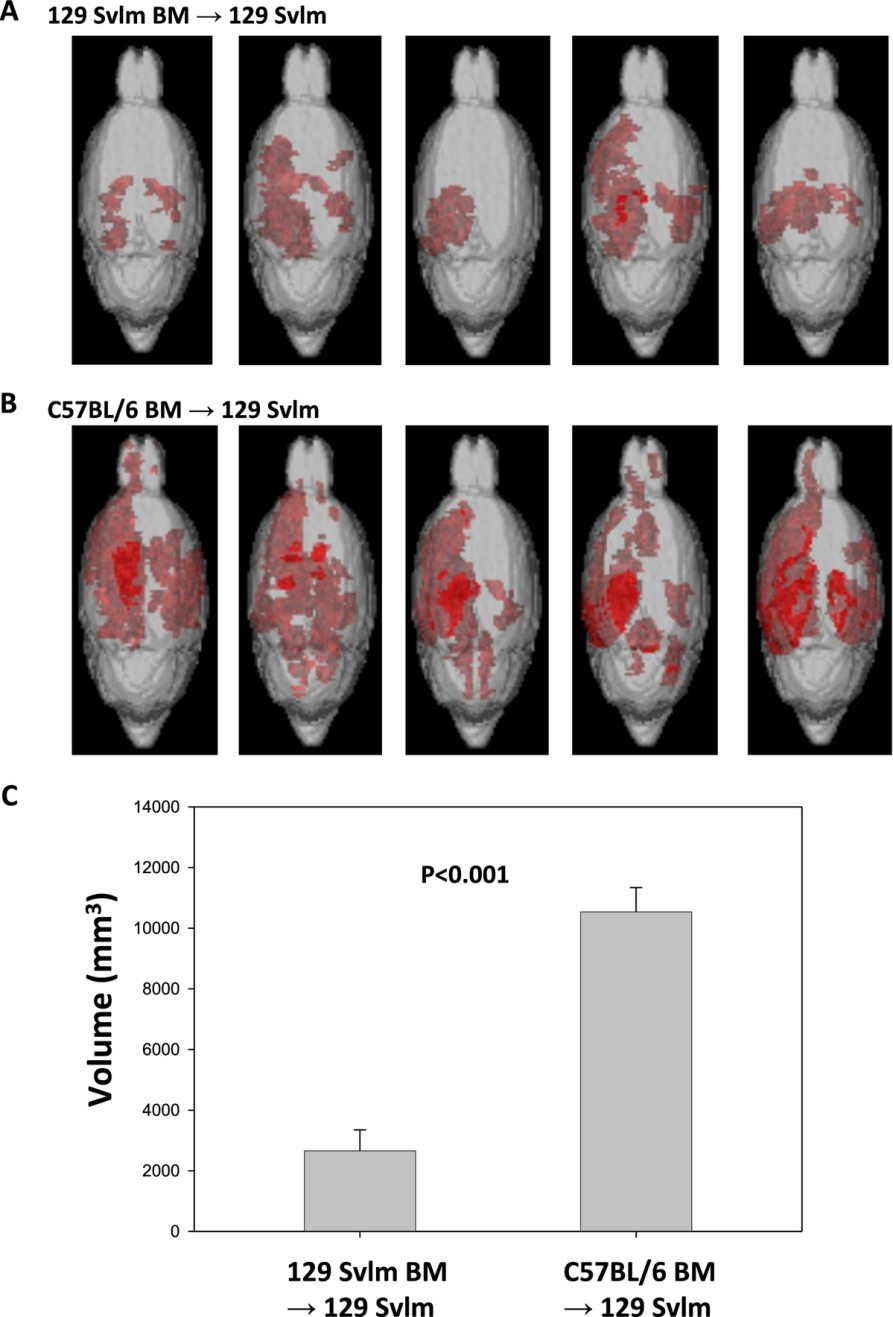
**129 Svlm mice reconstituted with C57BL/6 bone marrow display an increase in central nervous system vascular permeability as measured by gadolinium-enhanced T1-weighted MRI scans 24 hours post-VP2_121-130 _peptide administration to induce peptide-induced fatal syndrome**. The extent of central nervous system vascular permeability is shown by three-dimensional transparency rendering of gadolinium-enhancing areas in **(A) **129 Svlm mice reconstituted with autologous 129 Svlm bone marrow (*n *= 5) or in **(B) **129 Svlm mice reconstituted with C57BL/6 bone marrow (*n *= 5). Red areas indicate subvolumes with gadolinium enhancement. The overall thickness of underlying gadolinium enhancement and the distance from the surface influence the intensity of these areas. **(C) **Quantification of the three-dimensional volume of vascular permeability in each group. 129 Svlm mice reconstituted with C57BL/6 bone marrow exhibit a significant increase in vascular permeability compared to 129 Svlm mice reconstituted with autologous 129 Svlm bone marrow (*P *< 0.001).

The bone marrow transplant described above implied that a hematopoietic factor contributed to TMEV-infected animals' becoming moribund following intravenous injection with VP2_121-130 _peptide to induce PIFS. We also determined that C57BL/6 mice presented with significant astrocyte activation and increased T2*-weighted MRI lesions indicative of microhemorrhage formation following induction of PIFS compared to 129 SvIm mice. We therefore addressed the potential contribution of hematopoietic cells to astrocyte activation and microhemorrhage formation. 129 SvIm mice were lethally irradiated and then reconstituted either with autologous 129 SvIm bone marrow to serve as negative controls or with C57BL/6 mouse bone marrow. Eight weeks following reconstitution both groups were infected with TMEV and induced to undergo PIFS with administration of VP2_121-130 _peptide. 129 SvIm mice reconstituted with C57BL/6 bone marrow (*n *= 5) had significantly higher expression of GFAP in brain lysates compared to mice receiving 129 SvIm syngeneic bone marrow transfer (*n *= 12) (Figure 7). Seven of the 129 Svlm mice reconstituted with C57BL/6 bone marrow died before their brains were harvested to analyze lysates for GFAP expression. Similarly, 129 SvIm mice reconstituted with C57BL/6 bone marrow presented with high T2*-weighted MRI lesion loads, indicative of microhemorrhage formation (Figure 8, lower panels). In contrast, 129 SvIm mice that received autologous bone marrow had greatly reduced microhemorrhage formation (*P *= 0.006) (Figure 8, upper panels). Images were acquired for four animals from each group. These experiments demonstrate that hematopoietic factors expressed by the C57BL/6 mouse strain contribute to activation of astrocytes as determined by upregulation of GFAP and microhemorrhage formation. We have also shown that C57BL/6 mice exhibit higher levels of CNS vascular permeability than 129 Svlm mice as measured by FITC-albumin leakage into the CNS (Figure 4). To fully visualize and quantify the extent of CNS vascular leakage and determine whether a hematopoietic factor was contributing to this permeability, we performed bone marrow transplants and employed three-dimensional volumetric analysis of gadolinium-enhanced T1-weighted MRI studies using Analyze 10 software, which was developed by the Mayo Clinic's Biomedical Imaging Resource [45,46]. 129 Svlm mice reconstituted with C57BL/6 bone marrow (*n *= 5) displayed a significant increase in CNS vascular permeability after induction of PIFS (Figure 9A) compared to 129 Svlm mice reconstituted with autologous 129 Svlm bone marrow (*n *= 5) (*P *< 0.001) (Figure 9B). The quantification of the three-dimensional volume of gadolinium leakage from vasculature is shown in Figure 9C. Bone marrow transfer efficacy was approximately 75% as determined by fluorescence-activated cell-sorting analysis (data not shown). These results demonstrate that hematopoietic factors expressed by the C57BL/6 mouse strain also contribute to increased CNS vascular permeability following induction of PIFS.

## Discussion

In this study, we investigated the contribution of hematopoietic cells to antiviral CD8 T cell-initiated CNS vascular permeability. We previously demonstrated that C57BL/6 and 129 SvIm mouse strains, despite having highly similar CD8 T-cell responses, differed in susceptibility to PIFS following administration of VP2_121-130 _peptide. C57BL/6 mice were highly susceptible to PIFS, whereas 129 SvIm mice, which develop a similar CD8 T-cell response, were highly resistant to this fatal syndrome [27]. Resistance coincided with reduced glial cell activation and mouse survival [27]. We also observed a significant reduction in CNS vascular permeability leakage in 129 SvIm mice compared to C57BL/6 mice in this study among animals with PIFS. However, 129 SvIm mice did exhibit some measurable vascular permeability over mock peptide-administered controls. This result strongly implies that low levels of vascular permeability alone are not ultimately fatal. Rather, these results imply that more severe BBB disruption which coincides with microhemorrhage is what ultimately contributes to C57BL/6 mice becoming moribund. In particular, the microhemorrhaging observed in the hypothalamus and brainstem would contribute to the lethality of this condition. Therefore, we put forward a working model in which disruption of vascular integrity is fatal when it is associated with hemorrhage in vital regions of the CNS.

A central question pertaining to the onset of hemorrhage is the relative contributions of blood-derived factors versus stromal CNS tissue. We therefore sought to determine the extent to which susceptibility to PIFS is related to hematopoietic factors through bone marrow reconstitution. The results of the experiments put forward in this study demonstrate that functional deficit, the development of microhemorrhaging visible as hypointense lesions on T2*-weighted MRI scans, and the increase in CNS vascular permeability as seen through increased volumes of gadolinium leakage from vasculature on T1-weighted MRI scans are observed in normally PIFS-resistant 129 SvIm mice through reconstitution with C57BL/6 bone marrow. It is possible that the increase in microhemorrhage formation may be more severe in 129 Svlm mice that receive C57BL/6 bone marrow than in C57BL/6 mice alone, owing to the potential impact of irradiation, which has been shown to alter the BBB [47,48]. Nevertheless, irradiated 129 Svlm mice that receive C57BL/6 bone marrow display a significant increase in microhemorrhage formation compared to irradiated 129 Svlm mice that receive syngeneic bone marrow transfers. These observations support a hypothesis in which blood-derived cells, not CNS tissue, contribute to pathogenesis in the PIFS model of fatal immune-mediated BBB disruption and that the onset of hemorrhage plays an important role in this process.

Perforin expression is required for BBB disruption and the fatal syndrome initiated by D^b^: VP2_121-130 _epitope-specific CD8 T cells [24]. As put forward in this manuscript, equivalent perforin-mediated killing among CNS-infiltrating CD8 T cells was observed in both the C57BL/6 and 129 SvIm mouse strains. This observation demonstrates that, though perforin is critical for CD8 T-cell-initiated BBB disruption in the C57BL/6 mouse, additional modifying hematopoietic genes alter CNS vascular permeability and the ensuing fatal syndrome. One possibility is that the hematopoietic factors that affect CNS vascular permeability are delivered by the perforin pathway. In addition to initiating cytotoxicity in target cells, a more recently revealed role for perforin includes controlling virus replication in sensory neurons without killing them [49]. There are also an increasing number of inflammatory mediators delivered via cytotoxic granules in addition to granzyme B that could contribute to CNS vascular permeability [50]. A second possibility is that hematopoietic factors which contribute to BBB disruption are not related to perforin. In addition to our studies involving CD8 T cells and perforin, a role for BBB disruption by other immune cell types, including TH_17 _cells, monocytes and neutrophils, has also been put forward [21-23]. Class II gene-deficient mice are susceptible to PIFS, thus strongly implying that CD4 T-cell subsets are not required for this syndrome to occur [27]. Nevertheless, the contribution of these additional immune cell subsets will be further defined in this model system.

The list of potential hematopoietic factors in promoting BBB disruption is extensive. VEGF is one major candidate that may be an important mediator in CD8 T-cell-initiated BBB disruption [25,51]. We have shown in this study that VEGF is significantly increased in response to VP2_121-130 _peptide administration in 7-day TMEV-infected C57BL/6 mice, but not in 129 Svlm mice. This increase in VEGF may promote increased CNS vascular permeability, which is also seen in C57BL/6 mice induced to develop PIFS, but not in 129 Svlm mice. In support of this hypothesis, we previously showed that inhibiting VEGF causes a reduction in CNS vascular permeability and promotes survival [25]. Therefore, it will be essential to further investigate this potential mechanism of CD8 T-cell-initiated BBB disruption. Other potential factors that may contribute to BBB disruption are cytokines, including IL-2, TNF-α, lymphotoxins and IL-6 [7,52]. Various chemokines and matrix metalloproteases (MMPs) are additional inflammatory mediators reported to alter BBB permeability [53,54]. A role for death receptors in inducing a permeable state among endothelial cells through promoting upregulation of MMP9 in model systems has also been put forward [55]. Given the large number of candidate hematopoietic factors that could confer resistance and susceptibility to T-cell-mediated BBB disruption, the most robust approach to defining the critical genetic factors that contribute to CNS vascular permeability in the PIFS model will involve gene mapping. The experiments conducted in this study set the stage for such an analysis because rotarod performance, astrocyte activation and CNS vascular permeability are significantly different between the C57BL/6 and 129 SvIm mouse strains in this model. This model system will therefore enable a comprehensive genetic analysis to define quantitative trait loci that map to each of these characteristics.

## Conclusions

This study demonstrates the importance of the hematopoietic compartment in altering glial cell activation, vascular permeability and the onset of microhemorrhaging in the brain. This is significant because it demonstrates that the resulting fatal syndrome is not dictated by stromal tissue, but rather by blood-derived cells. Therefore, a concerted effort to define hematopoietic factors responsible for immune-mediated CNS vascular permeability will ultimately result in therapeutic strategies to suppress or induce opening of the BBB in neurological diseases.

## Abbreviations

BBB: blood-brain barrier; CTL: cytotoxic T lymphocyte; GFAP: glial fibrillary acidic protein; IL: interleukin; MMP: matrix metalloprotease; MRI: magnetic resonance imaging; PIFS: peptide-induced fatal syndrome; TMEV: Theiler's murine encephalomyelitis virus; TNF: tumor necrosis factor; VEGF: vascular endothelial growth factor.

## Competing interests

The authors declare that they have no competing interests.

## Authors' contributions

HLJ performed flow cytometry on neutrophils and CD8 T cells during acute TMEV, assisted with bone marrow transplants, performed rotarod behavioral assays, acquired and quantified T1-weighted MRI scans, constructed the figures and prepared the manuscript. YC performed Western blot analysis, rotarod behavioral assays and vascular permeability assays; constructed the figures; and prepared the manuscript. GLS performed Western blot analysis, rotarod behavioral assays and vascular permeability assays. JRM assisted with bone marrow transplants and optimization of flow cytometry. AKH performed flow cytometry, rotarod behavioral assays and quantification of MRI scans. LMH assisted with bone marrow transplants, rotarod behavioral assays and T1-weighted MRI acquisition. FJ assisted with Western blot analysis. IP optimized T1-weighted, gadolinium-enhanced and T2*-weighted MRI and was responsible for experiment design. AJJ was responsible for intravenous injections and experiment design and participated in manuscript preparation. All authors read and approved the final manuscript.

## Authors' information

AJJ is an associate professor in the Departments of Immunology and Neurology and specializes in neuroimmunology of the blood-brain barrier in viral infection, multiple sclerosis and neuro-oncology model systems.

## References

[B1] Zlokovic BV The blood-brain barrier in health and chronic neurodegenerative disorders Neuron 2008 57 178 201 10.1016/j.neuron.2008.01.003 18215617

[B2] Solomon T Dung NM Vaughn DW Kneen R Thao LT Raengsakulrach B Loan HT Day NP Farrar J Myint KS Warrell MJ James WS Nisalak A White NJ Neurological manifestations of dengue infection Lancet 2000 355 1053 1059 10.1016/S0140-6736(00)02036-5 10744091

[B3] Janssen HL Bienfait HP Jansen CL van Duinen SG Vriesendorp R Schimsheimer RJ Groen J Osterhaus AD Fatal cerebral oedema associated with primary dengue infection J Infect 1998 36 344 346 10.1016/S0163-4453(98)94783-1 9661954

[B4] Pancharoen C Mekmullica J Thisyakorn U Primary dengue infection: what are the clinical distinctions from secondary infection? Southeast Asian J Trop Med Public Health 2001 32 476 480 11944702

[B5] George R Lam SK Dengue virus infection: the Malaysian experience Ann Acad Med Singapore 1997 26 815 819 9522985

[B6] Shacklett BL Cox CA Wilkens DT Karlsson KR Nilsson A Nixon DF Price RW Increased adhesion molecule and chemokine receptor expression on CD8^+ ^T cells trafficking to cerebrospinal fluid in HIV-1 infection J Infect Dis 2004 189 2202 2212 10.1086/421244 15181567

[B7] Minagar A Alexander JS Blood-brain barrier disruption in multiple sclerosis Mult Scler 2003 9 540 549 10.1191/1352458503ms965oa 14664465

[B8] Kirk J Plumb J Mirakhur M McQuaid S Tight junctional abnormality in multiple sclerosis white matter affects all calibres of vessel and is associated with blood-brain barrier leakage and active demyelination J Pathol 2003 201 319 327 10.1002/path.1434 14517850

[B9] Plumb J McQuaid S Mirakhur M Kirk J Abnormal endothelial tight junctions in active lesions and normal-appearing white matter in multiple sclerosis Brain Pathol 2002 12 154 169 11958369 10.1111/j.1750-3639.2002.tb00430.x PMC8095734

[B10] Stone LA Smith ME Albert PS Bash CN Maloni H Frank JA McFarland HF Blood-brain barrier disruption on contrast-enhanced MRI in patients with mild relapsing-remitting multiple sclerosis: relationship to course, gender, and age Neurology 1995 45 1122 1126 7783875 10.1212/wnl.45.6.1122

[B11] Green R Scott LK Minagar A Conrad S Sepsis associated encephalopathy (SAE): a review Front Biosci 2004 9 1637 1641 10.2741/1250 14977574

[B12] Sharshar T Annane D de la Grandmaison GL Brouland JP Hopkinson NS Françoise G The neuropathology of septic shock Brain Pathol 2004 14 21 33 14997934 10.1111/j.1750-3639.2004.tb00494.x PMC8095740

[B13] Medana IM Turner GD Human cerebral malaria and the blood-brain barrier Int J Parasitol 2006 36 555 568 10.1016/j.ijpara.2006.02.004 16616145

[B14] Gibbs WN Kreidie MA Kim RC Hasso AN Acute hemorrhagic leukoencephalitis: neuroimaging features and neuropathologic diagnosis J Comput Assist Tomogr 2005 29 689 693 10.1097/01.rct.0000173843.82364.db 16163044

[B15] Oby E Janigro D The blood-brain barrier and epilepsy Epilepsia 2006 47 1761 1774 10.1111/j.1528-1167.2006.00817.x 17116015

[B16] Lacerda-Queiroz N Rodrigues DH Vilela MC Rachid MA Soriani FM Sousa LP Campos RD Quesniaux VF Teixeira MM Teixeira AL Platelet-activating factor receptor is essential for the development of experimental cerebral malaria Am J Pathol 2012 180 246 255 10.1016/j.ajpath.2011.09.038 22079430

[B17] de Vries NA Beijnen JH Boogerd W van Tellingen O Blood-brain barrier and chemotherapeutic treatment of brain tumors Expert Rev Neurother 2006 6 1199 1209 10.1586/14737175.6.8.1199 16893347

[B18] Blamire AM Anthony DC Rajagopalan B Sibson NR Perry VH Styles P Interleukin-1β-induced changes in blood-brain barrier permeability, apparent diffusion coefficient, and cerebral blood volume in the rat brain: a magnetic resonance study J Neurosci 2000 20 8153 8159 11050138 10.1523/JNEUROSCI.20-21-08153.2000 PMC6772751

[B19] Stoll G Jander S Schroeter M Cytokines in CNS disorders: neurotoxicity versus neuroprotection J Neural Transm Suppl 2000 59 81 89 10961421 10.1007/978-3-7091-6781-6_11

[B20] Chavarria A Alcocer-Varela J Is damage in central nervous system due to inflammation? Autoimmun Rev 2004 3 251 260 10.1016/j.autrev.2003.09.006 15246020

[B21] Kim JV Kang SS Dustin ML McGavern DB Myelomonocytic cell recruitment causes fatal CNS vascular injury during acute viral meningitis Nature 2009 457 191 195 10.1038/nature07591 19011611 PMC2702264

[B22] Kebir H Kreymborg K Ifergan I Dodelet-Devillers A Cayrol R Bernard M Giuliani F Arbour N Becher B Prat A Human T_H_17 lymphocytes promote blood-brain barrier disruption and central nervous system inflammation Nat Med 2007 13 1173 1175 10.1038/nm1651 17828272 PMC5114125

[B23] Argaw AT Gurfein BT Zhang Y Zameer A John GR VEGF-mediated disruption of endothelial CLN-5 promotes blood-brain barrier breakdown Proc Natl Acad Sci USA 2009 106 1977 1982 10.1073/pnas.0808698106 19174516 PMC2644149

[B24] Suidan GL McDole JR Chen Y Pirko I Johnson AJ Induction of blood brain barrier tight junction protein alterations by CD8 T cells PLoS One 2008 3 e3037 10.1371/journal.pone.0003037 18725947 PMC2516328

[B25] Suidan GL Dickerson JW Chen Y McDole JR Tripathi P Pirko I Seroogy KB Johnson AJ CD8 T cell-initiated vascular endothelial growth factor expression promotes central nervous system vascular permeability under neuroinflammatory conditions J Immunol 2010 184 1031 1040 10.4049/jimmunol.0902773 20008293 PMC2896014

[B26] Pirko I Suidan GL Rodriguez M Johnson AJ Acute hemorrhagic demyelination in a murine model of multiple sclerosis J Neuroinflammation 2008 5 31 10.1186/1742-2094-5-31 18606015 PMC2474604

[B27] Johnson AJ Mendez-Fernandez Y Moyer AM Sloma CR Pirko I Block MS Rodriguez M Pease LR Antigen-specific CD8^+ ^T cells mediate a peptide-induced fatal syndrome J Immunol 2005 174 6854 6862 15905527 10.4049/jimmunol.174.11.6854

[B28] Loke H Bethell DB Phuong CX Dung M Schneider J White NJ Day NP Farrar J Hill AV Strong HLA class I-restricted T cell responses in dengue hemorrhagic fever: a double-edged sword? J Infect Dis 2001 184 1369 1373 10.1086/324320 11709777

[B29] Zivna I Green S Vaughn DW Kalayanarooj S Stephens HA Chandanayingyong D Nisalak A Ennis FA Rothman AL T cell responses to an HLA-B*07-restricted epitope on the dengue NS3 protein correlate with disease severity J Immunol 2002 168 5959 5965 12023403 10.4049/jimmunol.168.11.5959

[B30] Mongkolsapaya J Dejnirattisai W Xu XN Vasanawathana S Tangthawornchaikul N Chairunsri A Sawasdivorn S Duangchinda T Dong T Rowland-Jones S Yenchitsomanus PT McMichael A Malasit P Screaton G Original antigenic sin and apoptosis in the pathogenesis of dengue hemorrhagic fever Nat Med 2003 9 921 927 10.1038/nm887 12808447

[B31] Green S Pichyangkul S Vaughn DW Kalayanarooj S Nimmannitya S Nisalak A Kurane I Rothman AL Ennis FA Early CD69 expression on peripheral blood lymphocytes from children with dengue hemorrhagic fever J Infect Dis 1999 180 1429 1435 10.1086/315072 10515800

[B32] Stephens HA Klaythong R Sirikong M Vaughn DW Green S Kalayanarooj S Endy TP Libraty DH Nisalak A Innis BL Rothman AL Ennis FA Chandanayingyong D HLA-A and -B allele associations with secondary dengue virus infections correlate with disease severity and the infecting viral serotype in ethnic Thais Tissue Antigens 2002 60 309 318 10.1034/j.1399-0039.2002.600405.x 12472660

[B33] Kurane I Innis BL Nimmannitya S Nisalak A Meager A Janus J Ennis FA Activation of T lymphocytes in dengue virus infections: high levels of soluble interleukin 2 receptor, soluble CD4, soluble CD8, interleukin 2, and interferon-γ in sera of children with dengue J Clin Invest 1991 88 1473 1480 10.1172/JCI115457 1939640 PMC295652

[B34] Skulina C Schmidt S Dornmair K Babbe H Roers A Rajewsky K Wekerle H Hohlfeld R Goebels N Multiple sclerosis: brain-infiltrating CD8^+ ^T cells persist as clonal expansions in the cerebrospinal fluid and blood Proc Natl Acad Sci USA 2004 101 2428 2433 10.1073/pnas.0308689100 14983026 PMC356967

[B35] Babbe H Roers A Waisman A Lassmann H Goebels N Hohlfeld R Friese M Schröder R Deckert M Schmidt S Ravid R Rajewsky K Clonal expansions of CD8^+ ^T cells dominate the T cell infiltrate in active multiple sclerosis lesions as shown by micromanipulation and single cell polymerase chain reaction J Exp Med 2000 192 393 404 10.1084/jem.192.3.393 10934227 PMC2193223

[B36] Johnson AJ Njenga MK Hansen MJ Kuhns ST Chen L Rodriguez M Pease LR Prevalent class I-restricted T-cell response to the Theiler's virus epitope D^b^:VP2_121-130 _in the absence of endogenous CD4 help, tumor necrosis factor α, γ interferon, perforin, or costimulation through CD28 J Virol 1999 73 3702 3708 10196262 10.1128/jvi.73.5.3702-3708.1999 PMC104145

[B37] Lin X Thiemann NR Pease LR Rodriguez M VP1 and VP2 capsid proteins of Theiler's virus are targets of H-2D-restricted cytotoxic lymphocytes in the central nervous system of B10 mice Virology 1995 214 91 99 10.1006/viro.1995.9951 8525642

[B38] McDole JR Danzer SC Pun RY Chen Y Johnson HL Pirko I Johnson AJ Rapid formation of extended processes and engagement of Theiler's virus-infected neurons by CNS-infiltrating CD8 T cells Am J Pathol 2010 177 1823 1833 10.2353/ajpath.2010.100231 20813972 PMC2947278

[B39] Johnson AJ Upshaw J Pavelko KD Rodriguez M Pease LR Preservation of motor function by inhibition of CD8+ virus peptide-specific T cells in Theiler's virus infection FASEB J 2001 15 2760 2762 11606479 10.1096/fj.01-0373fje

[B40] Kovacevic N Henderson JT Chan E Lifshitz N Bishop J Evans AC Henkelman RM Chen XJ A three-dimensional MRI atlas of the mouse brain with estimates of the average and variability Cereb Cortex 2005 15 639 645 15342433 10.1093/cercor/bhh165

[B41] Denic A Macura SI Mishra P Gamez JD Rodriguez M Pirko I MRI in rodent models of brain disorders Neurotherapeutics 2011 8 3 18 10.1007/s13311-010-0002-4 21274681 PMC3075741

[B42] Pirko I Gamez J Johnson AJ Macura SI Rodriguez M Dynamics of MRI lesion development in an animal model of viral-induced acute progressive CNS demyelination Neuroimage 2004 21 576 582 10.1016/j.neuroimage.2003.09.037 14980559

[B43] Pirko I Johnson AJ Chen Y Lindquist DM Lohrey AK Ying J Dunn RS Brain atrophy correlates with functional outcome in a murine model of multiple sclerosis Neuroimage 2011 54 802 806 10.1016/j.neuroimage.2010.08.055 20817104 PMC3858208

[B44] Pirko I Nolan TK Holland SK Johnson AJ Multiple sclerosis: pathogenesis and MR imaging features of T1 hypointensities in a murine model Radiology 2008 246 790 795 A published erratum appears in *Radiology *2008, **248:**322 10.1148/radiol.2463070338 18309014

[B45] Robb RA 3-D visualization in biomedical applications Annu Rev Biomed Eng 1999 1 377 399 10.1146/annurev.bioeng.1.1.377 11701494

[B46] Robb RA The biomedical imaging resource at Mayo Clinic IEEE Trans Med Imaging 2001 20 854 867 10.1109/42.952724 11585203

[B47] Rubin P Gash DM Hansen JT Nelson DF Williams JP Disruption of the blood-brain barrier as the primary effect of CNS irradiation Radiother Oncol 1994 31 51 60 10.1016/0167-8140(94)90413-8 8041898

[B48] van Vulpen M Kal HB Taphoorn MJB El Sharouni SY Changes in blood-brain barrier permeability induced by radiotherapy: implications for timing of chemotherapy? Oncol Rep 2002 9 683 688 12066192

[B49] Knickelbein JE Khanna KM Yee MB Baty CJ Kinchington PR Hendricks RL Noncytotoxic lytic granule-mediated CD8^+ ^T cell inhibition of HSV-1 reactivation from neuronal latency Science 2008 322 268 271 10.1126/science.1164164 18845757 PMC2680315

[B50] Smyth MJ Kelly JM Sutton VR Davis JE Browne KA Sayers TJ Trapani JA Unlocking the secrets of cytotoxic granule proteins J Leukoc Biol 2001 70 18 29 11435481

[B51] Weis SM Cheresh DA Pathophysiological consequences of VEGF-induced vascular permeability Nature 2005 437 497 504 10.1038/nature03987 16177780

[B52] Baluna R Vitetta ES Vascular leak syndrome: a side effect of immunotherapy Immunopharmacology 1997 37 117 132 10.1016/S0162-3109(97)00041-6 9403331

[B53] Engelhardt B Molecular mechanisms involved in T cell migration across the blood-brain barrier J Neural Transm 2006 113 477 485 10.1007/s00702-005-0409-y 16550326

[B54] Jin R Yang G Li G Molecular insights and therapeutic targets for blood-brain barrier disruption in ischemic stroke: critical role of matrix metalloproteinases and tissue-type plasminogen activator Neurobiol Dis 2010 38 376 385 10.1016/j.nbd.2010.03.008 20302940 PMC2862862

[B55] Wosik K Biernacki K Khouzam MP Prat A Death receptor expression and function at the human blood brain barrier J Neurol Sci 2007 259 53 60 10.1016/j.jns.2006.08.018 17395209

